# Krüppel-like factor 5 is a crucial mediator of intestinal tumorigenesis in mice harboring combined *Apc*^*Min *^and *KRAS*^*V*12 ^mutations

**DOI:** 10.1186/1476-4598-9-63

**Published:** 2010-03-18

**Authors:** Mandayam O Nandan, Amr M Ghaleb, Beth B McConnell, Nilesh V Patel, Sylvie Robine, Vincent W Yang

**Affiliations:** 1 Division of Digestive Diseases, Department of Medicine, Emory University School of Medicine, 201 Whitehead Research Building, 615 Michael Street, Atlanta, GA 30322, USA; 2 Institut Curie, 26 rue d'Ulm, 75248 Paris Cedex-05, France; 3 Department of Hematology and Medical Oncology, Emory University School of Medicine, 201 Whitehead Research Building, 615 Michael Street, Atlanta, GA 30322, USA

## Abstract

**Background:**

Both mutational inactivation of the adenomatous polyposis coli (*APC*) tumor suppressor gene and activation of the *KRAS *oncogene are implicated in the pathogenesis of colorectal cancer. Mice harboring a germline *Apc*^*Min *^mutation or intestine-specific expression of the *KRAS*^*V*12 ^gene have been developed. Both mouse strains develop spontaneous intestinal tumors, including adenoma and carcinoma, though at a different age. The zinc finger transcription factor Krüppel-like factor 5 (KLF5) has previously been shown to promote proliferation of intestinal epithelial cells and modulate intestinal tumorigenesis. Here we investigated the *in vivo *effect of *Klf5 *heterozygosity on the propensity of *Apc*^*Min*^/*KRAS*^*V*12 ^double transgenic mice to develop intestinal tumors.

**Results:**

At 12 weeks of age, *Apc*^*Min*^/*KRAS*^*V*12 ^mice had three times as many intestinal tumors as *Apc*^*Min *^mice. This increase in tumor number was reduced by 92% in triple transgenic *Apc*^*Min*^/*KRAS*^*V*12^/*Klf5*^+/- ^mice. The reduction in tumor number in *Apc*^*Min*^/*KRAS*^*V*12^/*Klf5*^+/- ^mice was also statistically significant compared to *Apc*^*Min *^mice alone, with a 75% decrease. Compared with *Apc*^*Min*^/*KRAS*^*V*12^, tumors from both *Apc*^*Min*^/*KRAS*^*V*12^/*Klf5*^+/- ^and *Apc*^*Min *^mice were smaller. In addition, tumors from *Apc*^*Min *^mice were more distally distributed in the intestine as contrasted by the more proximal distribution in *Apc*^*Min*^/*KRAS*^*V*12 ^and *Apc*^*Min*^/*KRAS*^*V*12^/*Klf5*^+/- ^mice. Klf5 levels in the normal-appearing intestinal mucosa were higher in both *Apc*^*Min *^and *Apc*^*Min*^/*KRAS*^*V*12 ^mice but were attenuated in *Apc*^*Min*^/*KRAS*^*V*12^/*Klf5*^+/- ^mice. The levels of β-catenin, cyclin D1 and Ki-67 were also reduced in the normal-appearing intestinal mucosa of *Apc*^*Min*^/*KRAS*^*V*12^/*Klf5*^+/- ^mice when compared to *Apc*^*Min*^/*KRAS*^*V*12 ^mice. Levels of pMek and pErk1/2 were elevated in the normal-appearing mucosa of *Apc*^*Min*^/*KRAS*^*V*12 ^mice and modestly reduced in Apc^Min^/*KRAS*^*V*12^/*Klf5*^+/- ^mice. Tumor tissues displayed higher levels of both Klf5 and β-catenin, irrespective of the mouse genotype from which tumors were derived.

**Conclusions:**

Results of the current study confirm the cumulative effect of *Apc *loss and oncogenic *KRAS *activation on intestinal tumorigenesis. The drastic reduction in tumor number and size due to *Klf5 *heterozygosity in *Apc*^*Min*^/*KRAS*^*V*12 ^mice indicate a critical function of KLF5 in modulating intestinal tumor initiation and progression.

## Background

Cancer is the result of deregulated cellular homeostasis and is typically characterized by increased proliferation and/or decreased apoptosis [1]. The mammalian intestinal epithelium is a continuously renewing system that is carefully orchestrated throughout life [2]. Several important signaling pathways are involved in maintaining intestinal epithelial homeostasis and include the Wnt, Notch, Eph/Ephrin, Hedgehog and bone morphogenetic protein (BMP) pathways [2]. It is well established that genetic perturbations in proliferation or differentiation of intestinal epithelial cells can lead to physiological changes which may aid in the development of colorectal cancer [3].

Specific mutations have been associated with colorectal carcinogenesis. *RAS *genes are one of the most frequently mutated oncogenes in human tumors and occur in approximately 50% of colon cancers [4,5]. There are three isoforms of the *RAS *gene, *KRAS, HRAS *and *NRAS *- however, a majority of human tumors possess mutations in the *KRAS *gene [3-8]. RAS is a membrane-bound protein that is activated by growth factors including epidermal growth factor (EGF) and platelet-derived growth factor (PDGF) [9]. Upon activation, RAS becomes attached to GTP and elicits a signaling cascade that induces cell proliferation [10]. *KRAS *gene is indispensible for normal embryonic survival - targeted homozygous deletion of the mouse K-*ras *gene resulted in embryonic lethality between E12.5 and term [11,12]. In contrast, homozygous deletions in mouse H-r *as *or N-*ras *gene did not result in any significant phenotypic or viability changes [12-14].

Loss of heterozygosity (LOH) with consequent inactivation of tumor suppressor genes has been causally implicated in colon cancer formation [8]. One of the best-characterized tumor suppressor genes in colon cancer is the adenomatous polyposis coli (*APC*) gene. APC is part of the Wnt signaling pathway that regulates intestinal epithelial cell proliferation. Inactivation of APC causes nuclear translocation of normally membrane-bound β-catenin and subsequent activation of the β-catenin/TCF4 complex with resultant increased proliferation [15-17]. Patients with familial adenomatous polyposis (FAP) harbor heritable mutations in the *APC *gene and spontaneously develop adenomatous polyps throughout their intestinal tracts at an early age [18,19]. The *APC *gene is also inactivated in greater than 80% of sporadic colorectal cancer [20]. An autosomal dominant mouse model of multiple intestinal neoplasia (Min) was developed in C57BL/6 mice upon ethylnitrosourea treatment [21]. This mouse strain carries a germline mutation in the mouse *Apc *gene, resulting in truncation of the protein at amino acid position 850 [22]. As a result, *Apc*^*Min *^mice exhibit a phenotype similar to that of FAP patients [22].

Krüppel-like factors (KLFs) are zinc finger-containing, Sp1-like transcription factors that are involved in diverse physiological processes including proliferation, differentiation and embryonic development [23,24]. In the intestine, Krüppel-like factor 5 (KLF5) is predominantly expressed in the proliferating crypt epithelial cells [25,26]. KLF5 is important for embryonic development since homozygous deletion of *Klf5 *in mice is embryonic lethal [27]. We previously demonstrated that KLF5 has a pro-proliferative effect in cultured cells and does so by activating cell cycle regulatory proteins such as cyclin D1, cyclin B1 and Cdc2 [28,29]. In addition, KLF5 has been shown to be an important mediator of the HRAS and KRAS oncogenic pathways [28,30] as well as the Wnt pathway [31]. Adenomas and carcinomas in mice that express oncogenic *KRAS*^*V*12 ^from the intestine-specific villin promoter have increased KLF5 expression [30]. In addition, we recently showed that adenoma formation in *Apc*^*Min *^mice was significantly abrogated when *Apc*^*Min *^mice were bred to mice heterozygous for *Klf5 *[32]. We further showed that KLF5 interacts with β-catenin and facilitates the nuclear localization and transcriptional activity of β-catenin [32]. These studies suggest that KLF5 is an essential mediator of intestinal tumorigenesis in the context of *Apc*^*Min *^mutation.

Since KLF5 has been shown to mediate the function of both APC and RAS, and mutations in *APC *and *KRAS *are common events in colorectal cancer, we examined the role of KLF5 in mediating intestinal tumor formation in mice compound for *Apc*^*Min *^and intestine-specific *KRAS*^*V*12 ^mutations in the current study.

## Results

### *Klf5 *heterozygosity reduces intestinal adenoma formation in *Apc*^*Min*^/*KRAS*^*V12 *^mice

**Figure 1:**
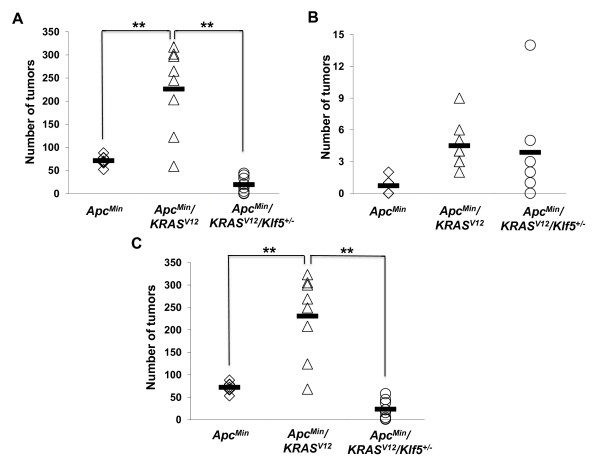
**The effect of *Klf5 *heterozygosity on intestinal tumor burden in *Apc*^*Min*^/*KRAS*^*V*12 ^mutant mice at 12 weeks of age**. Tumor burden is plotted as the number of tumors per mouse on the Y-axis. Tumor number from each individual mouse is shown as an open symbol, while the averages of the tumor numbers are represented as black bars. (A) Tumor burden in the small intestine; N = 8 for each genotype and **, *P *< 0.01. (B) Tumor burden in the colon; N = 8 for each genotype. (C) Overall tumor burden including both the small intestine and colon; N = 8 for each genotype and **, *P *< 0.01.

To determine the effect of *Klf5 *heterozygosity on intestinal adenoma formation in mice that harbor both *Apc*^*Min *^and *KRAS*^*V*12 ^mutations, we crossed mice that were heterozygous for the *Apc*^*Min *^and *Klf5 *genes with those that were heterozygous for the *KRAS*^*V*12 ^gene directed by the intestine-specific villin promoter [33]. Intestines from the resulting progeny were assessed for tumor number and size at 12 weeks of age. Tumors were observed in mice from three genotypes of the resulting progeny (*Apc*^*Min*^, *Apc*^*Min*^/*KRAS*^*V*12 ^and *Apc*^*Min*^/*KRAS*^*V*12^/*Klf5*^+/-^) but not in *Apc*^*Min*^/*Klf5*^+/- ^or *KRAS*^*V*12^mice. The mice with the compound *Apc*^*Min*^/*KRAS*^*V*12 ^genotype had a greater propensity for developing tumors in the small intestine than the *Apc*^*Min *^mice (Fig. 1A). The latter had an average of 71 small intestinal tumors per mouse while *Apc*^*Min*^/*KRAS*^*V*12 ^mice had an average of 226 tumors. The deletion of one of the *Klf5 *alleles in *Apc*^*Min*^/*KRAS*^*V*12 ^mice reduced the average tumor number to 19 per mouse - a 92% reduction (Fig. 1A). In the colon, the number of tumors per mouse was much fewer compared to the small intestine, with no significant differences in numbers of tumors between the three genotypes (Fig. 1B). Fig. 1C shows the combined tumor burden in both the small intestines and colons of the three different strains of mice.

### Haploinsufficiency of *Klf5 *decreases intestinal tumor size in Apc^*Min*^/*KRAS*^*V12 *^mice

**Figure 2:**
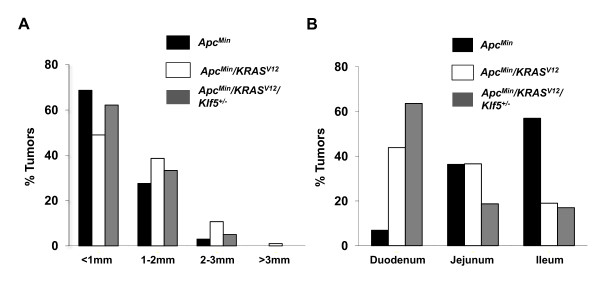
**Assessment of intestinal tumor size and distribution in mutant mice**. Percentages of intestinal tumors are displayed in bar graphs, with black bars representing *Apc*^*Min *^mice, white bars representing *Apc*^*Min*^/*KRAS*^*V*12 ^mice and gray bars indicating *Apc*^*Min*^/*KRAS*^*V*12^/*Klf5*^+/- ^mice. (A) Graph displaying tumor sizes in the small intestine. The tumors are sized based on 4 categories, <1 mm, 1-2 mm, 2-3 mm and >3 mm. Average percentages of tumors are represented on the Y-axis and tumor size categories on the X-axis; N = 8 and groups show a significant trend based on a one-way ANOVA test with *P *< 0.05. (B) Graph displaying tumor location in the small intestine. Tumor locations in the small intestine are divided into duodenum, jejunum and ileum. The graph is plotted with average percentage of tumors on the Y-axis and tumor locations on the X-axis; N = 8 and groups show a significant trend based on a one-way ANOVA test with *P *< 0.05.

In addition to tumor number, we measured the tumor size from the mice described above. The majority of the tumors, irrespective of genotype, were less than 1 mm in size (Fig. 2A). However, the percentage of tumors that were smaller than 1 mm in *Apc*^*Min*^/*KRAS*^*V*12 ^mice (49% overall) was lower than either *Apc*^*Min *^(69% overall) or *Apc*^*Min*^/*KRAS*^*V*12^/*Klf5*^+/- ^(62% overall) mice. In contrast, *Apc*^*Min*^/*KRAS*^*V*12 ^mice had a higher percentage of tumors that were 1-2 mm in size (39%) when compared to *Apc*^*Min*^/*KRAS*^*V*12^/*Klf5*^+/- ^mice (33%) or *Apc*^*Min *^mice (28%) (Fig. 2A). Similarly, *Apc*^*Min*^/*KRAS*^*V*12 ^mice also displayed a greater number of tumors that were 2-3 mm or greater than 3 mm when compared to the other two genotypes. These differences in tumor size showed a statistically significant trend when analyzed by the Chi-square test.

### Change in intestinal tumor localization in mice that possess the *KRAS*^*V12 *^genotype in addition to the *Apc*^*Min *^genotype

An interesting observation when comparing intestinal tumors among the different genotypes concerned the localization of the tumors. We observed that a larger percentage of tumors in *Apc*^*Min *^mice were localized in the distal small intestine, predominantly in the ileum (57%) and the jejunum (36%) (Fig. 2B). In contrast, both *Apc*^*Min*^/*KRAS*^*V*12 ^and *Apc*^*Min*^/*KRAS*^*V*12^/*Klf5*^+/- ^mice contained a higher percentage of intestinal tumors in the proximal small intestine, duodenum (44% and 64%, respectively) when compared to the *Apc*^*Min *^mice (7%) (Fig. 2B). These differences were found to be statistically significant using the Chi-square test.

**Figure 3:**
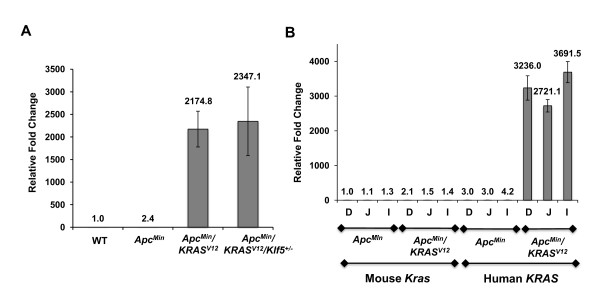
**Quantification of exogenous and endogenous *KRAS *transcript levels in the small intestine of mutant mice**. *KRAS *transcript levels were measured using quantitative PCR analysis. RNA was extracted from paraffin-embedded intestinal tissue samples. Endogenous (mouse) and exogenous (human) *KRAS *expression was measured and compared against β-actin. Fold changes were calculated for *KRAS *levels against β-actin levels using the 2^-ΔΔCt ^method of relative quantification [60]. (A) Relative fold changes in exogenous (human) KRAS transcript levels in mutant mice compared to the wild type (WT) mice (designated as 1). (B) Relative fold changes in mouse *Kras *and human *KRAS *transcript levels in different regions (D = duodenum; J = jejunum; I = ileum) of the mutant mouse intestines.

We then determined the level of *KRAS *transcripts in intestinal tissues from mice with the different genotypes using quantitative PCR. Both *Apc*^*Min*^/*KRAS*^*V*12 ^mice and *Apc*^*Min*^/*KRAS*^*V*12^/*Klf5*^+/- ^mice contained high levels of exogenous (human) *KRAS *mRNA in the intestine while wild type and *Apc*^*Min *^mice had only background expression (Fig. 3A). Since uneven *KRAS *expression could potentially contribute to the altered regional localization in the intestines of mice harboring *KRAS*^*V*12^, we measured both endogenous (mouse) and exogenous (human) *KRAS *transcript levels in different segments of the intestine. We found that levels of exogenous *KRAS *transcripts were highly elevated in all three segments of the intestine of *Apc*^*Min*^/*KRAS*^*V*12 ^mice, with no significant regional differences (Fig. 3B). Similarly, no regional differences in the levels of endogenous *Kras *were found in the intestines of either *Apc*^*Min *^or Apc^Min^/*KRAS*^*V*12 ^mice (Fig. 3B).

### *Klf5 *heterozygosity results in reduced levels of pro-proliferative proteins in the intestines of *Apc*^*Min *^and *Apc*^*Min*^/*KRAS*^*V12 *^mice

**Figure 4:**
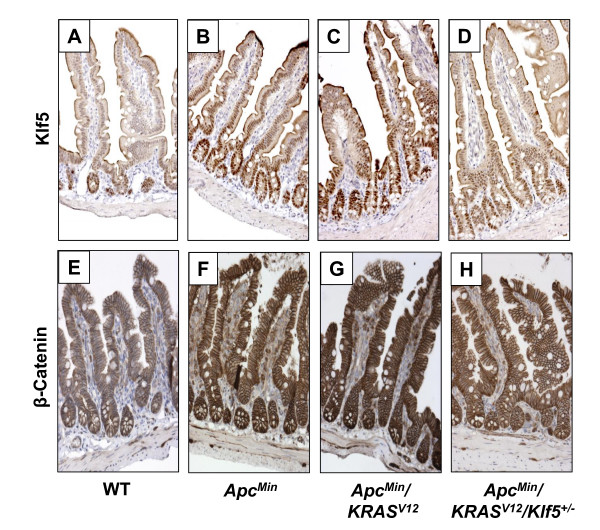
**Immunohistochemical analyses of Klf5 and β-catenin in the normal-appearing small intestines of wild type and mutant mice**. The panels are representative sections of normal-appearing small intestinal tissues stained with Klf5 (A-D) or β-catenin antibodies (E-H). Formalin-fixed, paraffin-embedded tissue sections (5 μm in size) were deparaffinized and antigen-retrieved using Citrate buffer (pH 6.0). Sections were stained with appropriate primary and secondary antibodies and developed using DAB chromogen. The resulting brown color is representative of protein expression. The sections were also counter stained with hematoxylin, which stains the nuclei blue. Panels A & E represent Klf5 and β-catenin staining, respectively, in wild type (WT) normal intestinal tissues. Panels B & F show staining in *Apc*^*Min *^tissues, while panels C & G and panels D & H show representative staining in *Apc*^*Min*^/*KRAS*^*V*12 ^and *Apc*^*Min*^/*KRAS*^*V*12^/*Klf5*^+/- ^tissues, respectively.

**Figure 5:**
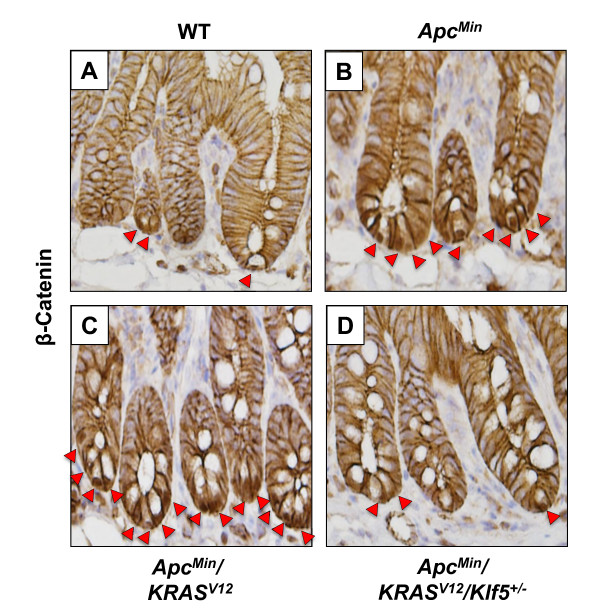
**Nuclear localization of β-catenin in the normal-appearing small intestines of wild type and mutant mice**. Panels are magnified immunohistochemical images of representative small intestinal crypts stained with β-catenin antibodies. Red arrowheads in all the panels indicate nuclear β-catenin staining.

We previously showed that KLF5 is pro-proliferative in the normal intestinal epithelial cells [30,34] and is increased in tumors from mice that contain the *Apc*^*Min *^allele [32] or the *KRAS*^*V*12 ^allele [30]. Here we observed increased levels of Klf5 protein in the normal-appearing small intestinal tissues of both *Apc*^*Min *^and *Apc*^*Min*^/*KRAS*^*V*12 ^mice when compared to that of wild type mice (Fig. 4A-C). The introduction of a mutant *Klf5 *allele into *Apc*^*Min*^/*KRAS*^*V*12 ^mice resulted in a reduction in Klf5 (Fig. 4D) to a level that was more similar to the wild type intestine (Fig. 4A). Similarly, the levels of β-catenin were increased in the normal-appearing intestinal tissues of *Apc*^*Min *^and *Apc*^*Min*^/*KRAS*^*V*12 ^mice when compared to wild type mice (Fig. 4E-G). Again, this increase in β-catenin was attenuated in the *Apc*^*Min*^/*KRAS*^*V*12^/*Klf5*^+/- ^mice (Fig. 4H). Moreover, an increase in nuclear localized β-catenin was noted in the crypt epithelial cells of *Apc*^*Min *^and *Apc*^*Min*^/*KRAS*^*V*12 ^mice compared to wild type mice (Fig. 5A-C). Similar to total β-catenin, the number of crypt epithelial cells containing nuclear β-catenin was reduced in *Apc*^*Min*^/*KRAS*^*V*12^/*Klf5*^+/- ^mice relative to *Apc*^*Min *^and *Apc*^*Min*^/*KRAS*^*V*12 ^mice (Fig. 5D). These results indicate that Klf5 modulates both steady-state β-catenin levels and cellular localization of β-catenin in intestinal epithelial cells secondary to the *Apc*^*Min *^mutation.

**Figure 6:**
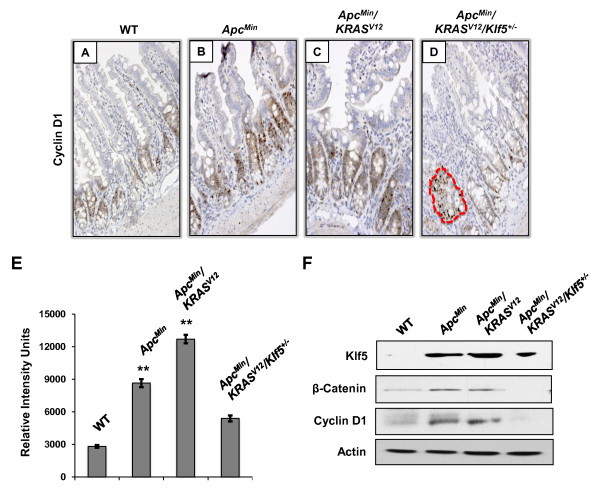
**Immunohistochemical and Western blot analyses of cyclin D1 in the normal-appearing small intestinal tissues of wild type and mutant mice**. (A-D) Immunohistochemical staining of cyclin D1 in the normal-appearing small intestines of wild type (WT) and mutant mice. A small focus of adenomatous tissue is demarcated by the red broken lines in panel D. (E) Quantification of cyclin D1 staining intensities in all fours sections using the Metamorph image analysis software. N = 10; **, P < 0.01. (F) Western blot analyses of Klf5, β-catenin, and cyclin D1 in the small intestines of wild type and mutant mice. Actin serves as a loading control.

**Figure 7:**
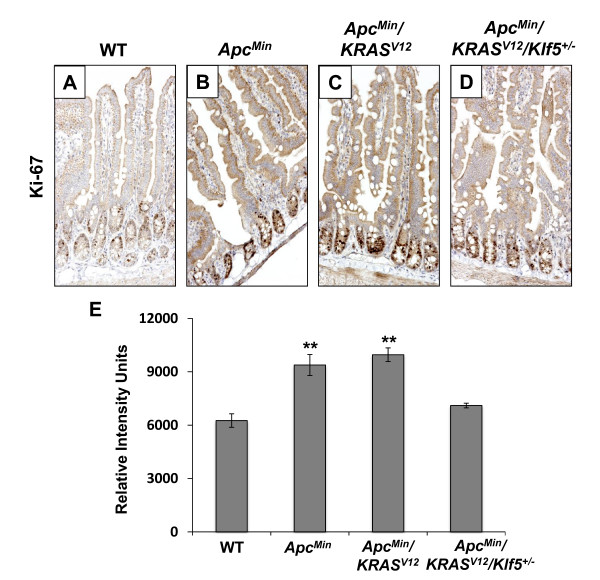
**Immunohistochemical analyses of Ki67 in the normal-appearing small intestinal tissues of wild type and mutant mice**. (A-D) Immunohistochemical staining of Ki67 in the normal-appearing small intestines of wild type (WT) and mutant mice. (E) Quantification of Ki67 cyclin D1 staining intensities in all fours sections using the Metamorph image analysis software. N = 10; **, P < 0.01.

We then performed immunohistochemical analyses on cyclin D1, a shared target between KLF5 and β-catenin [32]. Similar to the expression patterns of Klf5 and β-catenin, there was an increase in cyclin D1 levels in the intestine of both *Apc*^*Min *^and *Apc*^*Min*^/*KRAS*^*V*12 ^mice when compared to that of wild type mice (Fig. 6A-C). Cyclin D1 staining in the normal-appearing intestinal epithelium in *Apc*^*Min*^/*KRAS*^*V*12^/*Klf5*^+/- ^mice was reduced when compared to *Apc*^*Min *^and *Apc*^*Min*^/*KRAS*^*V*12 ^mice, except for a small focus of adenomatous tissue where cyclin D1 remained high (Fig. 6D). We also quantified cyclin D1 levels by quantitative image analysis (Fig. 6E) and Western blot analysis (Fig. 6F). As seen, both measurements confirmed the trend of cyclin D1 levels in the intestine from mice of the four genotypes as revealed by immunohistochemical staining. Similar trends in the levels of Klf5 and β-catenin were also documented by Western blot analysis (Fig. 6F). Lastly, levels of the proliferation marker, Ki67, in the normal-appearing intestinal tissues of the four strains of mice closely paralleled the levels of Klf5, β-catenin and cyclin D1, by immunohistochemical staining (Fig. 7A-D) and image quantification (Fig. 7E).

### The mitogen-activated kinase (MAPK) pathway is activated in the intestinal mucosa of *Apc*^*Min*^/*KRAS*^*V12 *^mice

**Figure 8:**
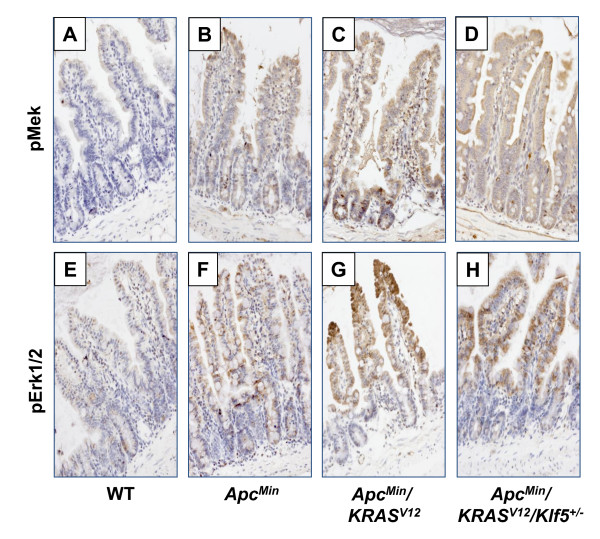
**Phosphorylation of MEK and ERK in the normal-appearing small intestinal tissues of wild type and mutant mice**. Immunohistochemical analyses were performed with phospho-Mek (pMek; A-D) and Phospho-Erk1/2 (pErk; E-H) antibodies. WT is wild type.

We previously established that MAPK pathway, as reflected by ERK phosphorylation, was an important intermediate in oncogenic KRAS-mediated induction of KLF5 [28,30]. Hence, we immunostained samples of small intestinal tissues for phospho-MEK and phospho-ERK proteins. We found that staining intensities for pMek were increased in normal-appearing small intestinal epithelial cells from both *Apc*^*Min *^and *Apc*^*Min*^/*KRAS*^*V*12 ^mice when compared to wild type mice (Fig. 8A-C). A moderate reduction in pMek staining was noted in the intestine of Apc^Min^/KRAS^V12^/Klf5^+/- ^mice compared to that of *Apc*^*Min*^/*KRAS*^*V*12 ^mice (Fig. 8C &8D). A similar pattern was also observed when pErk1/2 staining was performed (Fig. 8E-H). These results indicate that the MAPK pathway is activated in the intestine of *Apc*^*Min*^/*KRAS*^*V*12 ^mice and that *Klf5 *heterozygosity modestly reduces this activation.

### Intestinal tumors have increased Klf5 and β-catenin expression irrespective of genotype

**Figure 9:**
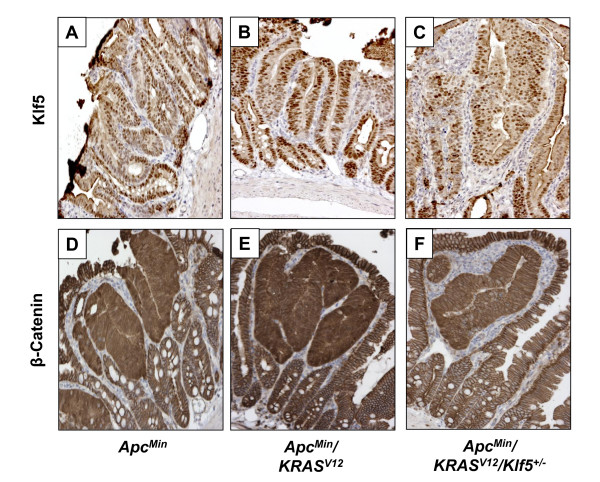
**Immunostaining of Klf5 and β-catenin in intestinal adenomas derived from mutant mice**. Adenomatous tissues from *Apc*^*Min*^, *Apc*^*Min*^/*KRAS*^*V*12 ^and *Apc*^*Min*^/*KRAS*^*V*12^/*Klf5*^+/- ^mice were formalin-fixed, paraffin-embedded and cut into 5 μm sections. Slides were then stained with Klf5 and β-catenin antibodies after deparaffinization and antigen-retrieval. Protein expression was determined upon secondary antibody treatment and color development using DAB chromogen (brown stain). Nuclei were then counterstained blue using hematoxylin. Panels A & D represent Klf5 and β-catenin staining in *Apc*^*Min *^tumor tissues. Panels B & E represent comparative staining in *Apc*^*Min*^/*KRAS*^*V*12 ^tumor tissues, while panels C & F represent staining in *Apc*^*Min*^/*KRAS*^*V*12^/*Klf5*^+/- ^tumor tissues.

We also stained intestinal tumors derived from *Apc*^*Min*^, *Apc*^*Min*^/*KRAS*^*V*12 ^and *Apc*^*Min*^/*KRAS*^*V*12^/*Klf5*^+/- ^mice for Klf5 and β-catenin. As seen in Fig. 9, the levels of both Klf5 and β-catenin were elevated in the adenomatous tissues of all three strains compared to the normal-appearing intestinal tissues. These results indicate that despite the differences in expression among proliferative markers in the normal intestinal epithelia of the mutant mice, expression patterns of these markers are similar in tumor tissues irrespective of genotype.

## Discussion

Colorectal cancer is the result of cumulative mutations in genes involved in regulating proliferation or apoptosis. APC is an integral part of the Wnt signaling pathway that regulates intestinal epithelial homeostasis [35]. Inactivation of *APC *is synonymous with Wnt activation and has been shown to be causal to colorectal carcinogenesis [35]. Also, among the frequently mutated genes in colorectal cancer is *KRAS*, specifically in codons 12, 13 and 61 [36-39]. It was shown that mutations in *APC *and *KRAS *occur in approximately 80% and 50%, respectively, of sporadic colorectal cancer [4,5,20]. Recent studies aimed at comprehensive sequencing of genes mutated in colorectal cancer confirmed that *APC *and *KRAS *mutations are among the most common mutations found in colorectal cancer [40,41].

Results of our study confirmed the cooperative effect of activated Wnt and RAS signaling in mice. At 12 weeks of age, compound heterozygous *Apc*^*Min*^/*KRAS*^*V*12 ^mice developed more and larger small intestinal tumors than *Apc*^*Min *^mice alone (Figs. 1A and 2A). In comparison, at the same age, *KRAS*^*V*12 ^mice did not have any tumor, consistent with the previous finding that these mice develop intestinal tumors relatively late in life [33]. This cooperative nature between *Apc *and *KRAS *mutations in leading to increased tumor formation is similar to that observed in two previous studies, one involving *Apc*^+/1638^/*KRAS*^*V*12 ^double transgenic mice [42] and the other *Apc*^*Min*^/K-*ras*^*D*12 ^double transgenic mice [43].

While there was a trend for a higher number of colonic tumors in the *Apc*^*Min*^/*KRAS*^*V*12 ^as compared to *Apc*^*Min *^mice alone in our study (Fig. 1B), the difference did not reach statistical significance, due to the relatively small number of tumors in this region. The propensity for the *Apc*^*Min*^, *Apc*^+/1638^, *KRAS*^*V*12^, *Apc*^+/1638^/*KRAS*^*V*12 ^mice to develop tumors in the small intestine rather than the colon has been reported [21,33,42]. It is of interest to note that there is a difference in regional distribution of small bowel tumors between *Apc*^*Min *^and *Apc*^*Min*^/*KRAS*^*V*12 ^mice - tumors in the former mice were more distally distributed while those in the latter were more proximally distributed (Fig. 2B). This difference in tumor distribution does not appear to be due to regional variations in expression of the *KRAS*^*V*12 ^transgene from the villin promoter (Fig. 3B). The effect of *KRAS*^*V*12 ^allele introduction on the shift in tumor distribution more proximally is therefore not clear at this time. A similar trend toward distribution of small bowel tumors in the *Apc*^*Min *^mice has been reported [44].

We recently reported the critical role for Klf5 in tumor initiation in *Apc*^*Min *^mice [32]. *Klf5 *haploinsufficiency in *Apc*^*Min *^mice resulted in a significant decrease in tumor number and size [32]. Results of the current study demonstrate a similar effect on tumor formation at 12 weeks of age in *Apc*^*Min*^/*KRAS*^*V*12 ^mice that were heterozygous for the *Klf5 *alleles, with the intestinal tumor burden reduced by more than 90% in the triple *Apc*^*Min*^/*KRAS*^*V*12^/*Klf5*^+/- ^transgenic mice when compared to the double *Apc*^*Min*^/*KRAS*^*V*12 ^transgenic mice (Fig. 1). In addition, the tumors in the *Apc*^*Min*^/*KRAS*^*V*12^/*Klf5*^+/- ^mice, when formed, were smaller than those from the *Apc*^*Min*^/*KRAS*^*V*12 ^mice (Fig. 2A). Indeed, *Apc*^*Min*^/*KRAS*^*V*12 ^mice had to be euthanized by 12 weeks of age, due to the presence of rectal prolapse from the large tumor burden. In contrast, the majority of *Apc*^*Min*^/*KRAS*^*V*12^/*Klf5*^+/- ^mice survived up to a year without displaying overt morbidity. Taken into consideration that expression of the *KRAS*^*V*12 ^transgene in the small intestine of *Apc*^*Min*^/*KRAS*^*V*12^/*Klf5*^+/- ^mice remains robust (Fig. 3A), our study suggests that haploinsufficiency of *Klf5 *attenuates the cumulative effect of *Apc *inactivation and oncogenic *KRAS *activation.

Our results show that a combined effect of *Apc*^*Min *^and *KRAS*^*V*12 ^mutations is a significant increase in the levels of β-catenin, cyclin D1 and Ki67, in the normal-appearing intestinal tissues in the *Apc*^*Min*^/*KRAS*^*V*12 ^mice as compared to wild type mice (Figs. 4, 5, 6, 7). This increase is similar to that seen in the intestine from the *Apc*^*Min *^mice (Figs. 4, 5, 6, 7). Haploinsufficiency of *Klf5 *attenuated the increase in the levels of these three proteins in the normal-appearing intestine of *Apc*^*Min*^/*KRAS*^*V*12 ^mice to levels that resembled the wild type intestine (Figs. 4, 5, 6, 7). These results indicate that the increase in β-catenin and cyclin D1 levels in the intestine of mutant mice is primarily a consequence of *Apc*^*Min *^mutation, rather than *KRAS*^*V*12 ^over-expression and that the tumor suppressive effect of *Klf5 *haploinsufficiency in *Apc*^*Min*^/*KRAS*^*V*12 ^mice is due primarily to the ability of Klf5 to modulate *Apc*^*Min *^signaling. These notions are supported by the observation that increased nuclear localization of β-catenin is observed in the normal-appearing intestinal crypt epithelial cells of both *Apc*^*Min *^and *Apc*^*Min*^/*KRAS*^*V*12 ^mice but was significantly reduced in the crypt cells of *Apc*^*Min*^/*KRAS*^*V*12^/*Klf5*^+/- ^mice (Fig. 5). The *se *findings are consistent with our previous observation that Klf5 both stabilizes β-catenin and facilitates nuclear import of β-catenin [32]. However, it should be noted that a recent report showed that activated *KRAS *also facilitates nuclear translocation of β-catenin following loss of *Apc *in zebrafish [45]. Moreover, we have shown that KRAS^V12 ^increases KLF5 expression *in vitro *and *in vivo *[30]. Combining the results of these studies, it is highly plausible that KLF5 is a common mediator for the increased β-catenin activity due to both *APC *loss and *KRAS *activation.

MEK and ERK phosphorylation are hallmarks of activation of the RAS signaling pathway which stimulates cell proliferation [46]. We previously reported that MEK/ERK phosphorylation is essential for mediating oncogenic RAS-induced *KLF5 *expression *in vitro *[28,30]. Previous studies have documented enhanced MEK/ERK protein phosphorylation in mice containing both oncogenic *KRAS *mutations and *Apc *inactivation [47,48]. Results of the current study showed a similar increase in MEK/ERK phosphorylation in the normal-appearing intestines of mice with *Apc*^*Min *^mutation that is further enhanced upon oncogenic *KRAS *activation (Fig. 8). Upon heterozygous loss of *Klf5 *in *Apc*^*Min*^/*KRAS*^*V*12 ^mice, MEK/ERK phosphorylation levels are only modestly reduced. These results suggest that RAS activation of MEK/ERK phosphorylation is upstream of *KLF5 *induction, although KLF5 could potentially regulate MEK/ERK phosphorylation through a feedback mechanism, as previously proposed [49].

Our study adds to a growing list of literature demonstrating the combined effect of *Apc *and *KRAS *mutation on intestinal tumorigenesis in mice [42,43,50,51]. In the setting of *Apc *mutation, inhibition of intestinal tumor formation has been documented secondary to deletion of several genes crucial for tumorigenesis [32,52-56]. However, ours is the first in which to show a critical role of Klf5 in mediating the tumorigenic effect of combined *Apc *and *KRAS *mutations, a commonly encountered scenario in colorectal cancer in humans. This suggests that therapies targeted to KLF5 may have potential therapeutic benefit to patients with colorectal cancer. Indeed, a recent screen for small molecule inhibitors of KLF5 expression has yielded several potent compounds that inhibit proliferation of colorectal cancer cells [57]. Further investigation may prove KLF5 an attractive target for intervention in the prevention or treatment of colorectal cancer.

## Conclusions

Loss of tumor suppressor genes and activation of oncogenes are hallmarks of cancers. In the case of colorectal cancer, loss of *APC *and activation of *KRAS *are common. Here, we present a robust mouse model of intestinal tumorigenesis with the generation of *Apc*^*Min*^/*KRAS*^*V*12 ^mice. These mice display an increased propensity for developing intestinal tumors at an early age compared to *Apc*^*Min *^mice. Moreover, we were able to significantly reduce tumor burden and size in the compound *Apc*^*Min*^/*KRAS*^*V*12 ^mice by reducing expression of *Klf5 *with genetic means. *Apc*^*Min*^/*KRAS*^*V*12^/*Klf5*^+/- ^mice display reduced levels of Klf5 protein as well as β-catenin, cyclin D1 and Ki67, all known markers of proliferation and transformation. We conclude that Klf5 is a crucial mediator of initiation and progression of intestinal tumors resulted from *Apc*^*Min *^and *KRAS*^*V*12 ^mutations.

## Methods

### Reagents

Antibodies used in the experiments were previously described [30,32]. Antibodies against KLF5 were generated against a synthetic KLF5 peptide in rabbits (Strategic Diagnostics, Newark, DE). Anti-KLF5 antibody was used at a dilution of 1:15,000 for immunohistochemistry and at 1:4,000 for Western blot analysis. Mouse monoclonal antibody against total β-catenin was purchased from Invitrogen (Carlsbad, CA) and used at a dilution of 1:1,000 for Western blot analyses. For immunohistochemical analysis, total β-catenin antibodies purchased from BD Biosciences (San Jose, CA) were used at 1:250 dilutions. Rabbit monoclonal cyclin D1 antibodies were purchased from Biocare Medical (Concord, CA) and used at 1:200 dilutions in immunohistochemical analyses and 1:2,500 dilutions for Western blot analysis. Anti-Ki67 antibodies were purchased from Novocastra (Leica Microsystems, Bannockburn, IL) and used at 1:500 dilutions. Anti-Phospho-MEK1 and anti-Phospho-ERK1/2 antibodies, used at 1:100 dilutions, were purchased from Cell Signaling Technology (Danvers, MA).

### Mice

All studies involving mice have been approved by the Emory University Institutional Animal Care and Use Committee (IACUC). C57BL/6J mice heterozygous for *KRAS*^*V*12 ^expressed from a mouse villin promoter were previously generated [33]. Mice double heterozygous for *Apc*^*Min *^and *Klf5*^+/- ^alleles were generated as previously described [32]. Founder C57BL/6J mice that were heterozygous *Apc*^*Min *^alleles (males) were mated with those that were heterozygous for *Klf5*^+/- ^alleles (females). The resulting progeny generated double heterozygous *Apc*^*Min*^/*Klf5*^+/- ^mice. These mice were then mated with the *KRAS*^*V*12 ^mice to generate the triple transgenic mice used in this study. Littermates of the crosses consisted of mice wild type for all alleles, mice that were heterozygous for only one of the three alleles, mice with two heterozygous alleles and mice with all three heterozygous alleles. Out of this progeny wild type, *Apc*^*Min*^, *Apc*^*Min*^/*KRAS*^*V*12 ^and *Apc*^*Min*^/*KRAS*^*V*12^/*Klf5*^+/- ^mice were used for the study.

### Genotype analyses

Genotype analyses were performed as previously described [58]. Tail-tips from newly weaned mice were collected and processed using the Red Extract-N-Amp kit as per protocol (Sigma Aldrich, St. Louis, MO). Allele-specific PCR analyses were performed using 2 μl of mouse DNA and appropriate primers for genotypic analyses. Primers to identify KLF5, *Apc*^*Min *^mutation, and villin-KRAS have been previously described [33,58,59].

### Tumor assessment

Mice were sacrificed at 12 weeks of age by CO_2 _asphyxiation, as per IACUC guidelines. The mice were dissected and the small intestine and colon removed. The intestinal tissues were cleaned with phosphate-buffered saline (PBS) and cut open. Using a dissecting microscope, the intestinal tissues were examined in a blinded fashion, for the presence and size measurements of tumors. The adenomas found were counted and measured according to <1 mm, 1-2 mm, 2-3 mm and >3 mm size groups.

### RNA purification and quantitative PCR

RNA was extracted from formalin-fixed paraffin-embedded tissue samples using the RT^2 ^FFPE RNA extraction kit (SA Biosciences, Frederick, MD). Sixty μm tissue sections were cut from paraffin sample blocks and digested with Proteinase K for 30 minutes. Samples were then boiled and centrifuged to remove paraffin. RNA was extracted from the liquid samples using Trizol LS reagent (Invitrogen, Carlsbad, CA) and subsequently purified using a spin column. RNA was quantified and used (100 ng/sample) in quantitative PCR. Specific primers against mouse KRas, human KRAS and mouse β-actin were purchased from SA Biosciences (Frederick, MD) and Qiagen (Valencia, CA) respectively. Quantitative PCR was performed using the *Power *SYBR Green RNA-to-C_T _*1-Step *kit (Invitrogen, Carlsbad, CA) as per protocol. Observed C_T _levels were then used to calculate fold change using the 2^-ΔΔCt ^method of relative quantification [60].

### Immunohistochemistry

Immunohistochemical analysis was performed as previously described [30]. Intestinal tissues dissected from mice were fixed overnight with 10% formalin buffer (Thermo Fisher Scientific, Fair Lawn, NJ). The tissues were then paraffinized using a tissue paraffinizer (Shandon Excelsior and Histocenter, Thermo Scientific, NJ). The paraffinized tissues were embedded onto paraffin blocks and cut into 5 μm sections using a microtome (Microm, Thermo Scientific, NJ). The sections were then dried onto charged slides and used for staining. The slides containing paraffin-embedded tissue sections were deparaffinized by baking in a 60°C oven for 1 hr and subsequent incubation in a xylene bath. Sections were incubated in a 5% hydrogen peroxide bath to block endogenous tissue peroxidases. The sections were then hydrated by incubation in a decreasing alcohol bath series (100%, 95%, 70%) followed by antigen retrieval in citrate buffer solution (10 mM Sodium citrate, 0.05% Tween-20, pH 6.0) at 125°C for 10 min using a decloaking chamber (Biocare Medical, CA). Tissue sections were then incubated with blocking buffer containing avidin (2% milk, 0.05% Tween-20, 5% normal serum) for 30 min at 37°C. Subsequently, antibodies, with Biotin, were added to the blocking buffer at appropriate concentrations and incubated with tissue sections for 1 hr at 37°C. Sections were washed and incubated with secondary antibodies at the appropriate concentration for 30 min at 37°C. Vectorstain ABC solution (Vector Labs, Burlingame, CA) and Betazoid DAB (Biocare Medical, CA) were used to reveal staining in tissues. The sections were then incubated in Gill's Hematoxylin (Vector Labs, CA), dehydrated and cover-slipped for observation. Slides were observed under a Zeiss Axioskop (Carl Zeiss MicroImaging, Thornwood, NY) and representative pictures taken.

### Quantification of immunohistochemical staining intensity

Staining intensities for immunohistochemical analyses were quantifies using Metamorph image analysis software (Version 7.1.1) (Molecular Devices, Downington, PA). Individual images were specifically quantified as previously described [30].

### Western blot analyses

Western blot analyses were performed as previously described [28]. Proteins were extracted from 20 μm paraffin embedded tissue sections using a previously established protocol [61]. Tissue sections were deparaffinized using xylene with the addition of 7.5% methanol. Samples were then centrifuged and the pellet dried in a fume hood for 3 min. The pellets were then resuspended in 20 mM Tris-HCl (pH 7.5) containing 2% SDS and the suspension heated in a 100°C heat block for 20 min. Subsequently, the samples were incubated in a 60°C oven for 2 hr. Protein content was measured and equal amounts of samples were loaded onto Bis-Tris gels (Invitrogen, CA). Proteins were transferred to nitrocellulose membranes (BioRad, Hercules, CA) and probed with appropriate primary antibodies. Blots were then washed and secondary antibodies applied at appropriate concentrations. Protein bands were then visualized on film upon chemiluminescent detection.

### Statistical analysis

A one-way ANOVA was used to compare mean numbers of tumors between *Apc*^*Min*^, *Apc*^*Min*^/*KRAS*^*V*12^, and *Apc*^*Min*^/*KRAS*^*V*12^/*Klf5*^+/- ^mice given independence of samples, equality of variances as tested by Levene's test, and a Gaussian distribution of the data. Multiple pair wise comparisons were made among groups using Tukey's test. Tumors were categorized based on size into 4 ordinal categories (<1 mm, 1-2 mm, 2-3 mm, and greater than 3 mm) using previously published measurement protocols [30]. Proportions of tumors among size categories were compared between *Apc*^*Min*^, *Apc*^*Min*^/*KRAS*^*V*12^, and *Apc*^*Min*^/*KRAS*^*V*12^/*Klf5*^+/- ^mice using a Chi-square test for homogeneity. *P *< 0.05 was considered indicative of statistical significance. Similar methods were used to ascertain statistical significance in relation to tumor location. The statistical software package SAS 9.2 was used for statistical analysis.

## Competing interests

The authors declare that they have no competing interests.

## Authors' contributions

MON and VWY conceived the design of the study and participated in drafting the manuscript. MON performed the immunohistochemical and Western blot analyses. AMG and MON were involved in the assessment of tumor burden and sizing from mice. BBM and AMG helped in providing transgenic mice and with the setup of immunohistochemical analyses. NVP performed statistical analyses on mice data. SR provided critical reagents and advised on the study design. All authors read and approved the final manuscript.
